# Medial Temporal Lobe Projections to the Retrosplenial Cortex of the Macaque Monkey

**DOI:** 10.1002/hipo.22024

**Published:** 2012-04-23

**Authors:** John P Aggleton, Nicholas F Wright, Seralynne D Vann, Richard C Saunders

**Affiliations:** 1 School of Psychology, Cardiff UniversityCardiff, Wales, United Kingdom CF10 3AT; 2 Laboratory of Neuropsychology, National Institute of Mental HealthBethesda, Maryland

**Keywords:** amygdala, cingulate cortex, entorhinal cortex, hippocampus, subiculum

## Abstract

The projections to the retrosplenial cortex (areas 29 and 30) from the hippocampal formation, the entorhinal cortex, perirhinal cortex, and amygdala were examined in two species of macaque monkey by tracking the anterograde transport of amino acids. Hippocampal projections arose from the subiculum and presubiculum to terminate principally in area 29. Label was found in layer I and layer III(IV), the former seemingly reflecting both fibers of passage and termination. While the rostral subiculum mainly projects to the ventral retrosplenial cortex, mid and caudal levels of the subiculum have denser projections to both the caudal and dorsal retrosplenial cortex. Appreciable projections to dorsal area 30 [layer III(IV)] were only seen following an extensive injection involving both the caudal subiculum and presubiculum. This same case provided the only example of a light projection from the hippocampal formation to posterior cingulate area 23 (layer III). Anterograde label from the entorhinal cortex injections was typically concentrated in layer I of 29a–c, though the very caudal entorhinal cortex appeared to provide more widespread retrosplenial projections. In this study, neither the amygdala nor the perirhinal cortex were found to have appreciable projections to the retrosplenial cortex, although injections in either medial temporal region revealed efferent fibers that pass very close or even within this cortical area. Finally, light projections to area 30V, which is adjacent to the calcarine sulcus, were seen in those cases with rostral subiculum and entorhinal injections. The results reveal a particular affinity between the hippocampal formation and the retrosplenial cortex, and so distinguish areas 29 and 30 from area 23 within the posterior cingulate region. The findings also suggest further functional differences within retrosplenial subregions as area 29 received the large majority of efferents from the subiculum. © 2012 Wiley Periodicals, Inc.

## INTRODUCTION

Research into the functions of the human retrosplenial cortex (areas 29 and 30) consistently suggests roles in both memory and navigation (Maguire,2001a, b; Vann et al.,^2009^). Evidence comes from descriptions of amnesia and topographic disorientation following pathology in the posterior cingulate region involving the retrosplenial cortex (Valenstein et al.,^1987^; Takahashi et al.,^1997^; Gainotti et al.,^1998^; Maguire,2001b; Greene et al.,^2006^; Ino et al.,^2007^). Other supportive evidence comes from functional magnetic resonance imaging (fMRI) studies (e.g., Maguire,2001a; Epstein et al.,^2007^; Iaria et al.,^2007^). To understand how this region might support aspects of memory and navigation, it is important to appreciate its connectivity and, in particular, how it is related with medial temporal lobe regions already implicated in similar functions. Anatomical tracing studies in monkeys offer a means to advance our understanding of these connections in the primate brain.

Neuroanatomical studies of macaque monkeys show that the retrosplenial cortex has connections consistent with its putative roles in memory and navigation. In particular, the retrosplenial cortex is reciprocally linked with the medial temporal lobe, limbic thalamic nuclei, and parietal cortex (area 7), as well as the dorsolateral and medial prefrontal cortices (Rosene and Van Hoesen,^1977^; Vogt and Pandya,^1987^; Vogt et al.,^1987^; Morris et al.,1999a, b; Kobayashi and Amaral,^2003^, ^2007^; Shibata and Yukie, 2003,^2009^; Yukie and Shibata,^2009^). Of these connections, those with themedial temporal lobe are most readily linked to memory (Sutherland and Hoesing,^1993^; Maguire,2001a, b; Vann et al.,^2009^).

A number of studies have used retrograde markers to show that the monkey retrosplenial cortex receives appreciable inputs from the subiculum, presubiculum, and parasubiculum (Baleydier and Mauguiere,^1980^; Vogt and Pandya,^1987^; Morris et al.,1999a; Kobayashi and Amaral,^2003^; Parvizi et al.,^2006^). These studies have, however, struggled to place discrete tracer injections within just area 29 or just area 30, and so the relative patterns of projections to these two areas remains to be confirmed. The only previous anterograde tracer study (Rosene and Van Hoesen,^1977^) simply noted the existence of subicular projections to the retrosplenial region, i.e., no further description was provided. To address this shortcoming, this study examined the axonal transport of radioactive amino acid tracers from the hippocampal formation (subiculum) to the retrosplenial cortex in a series of macaque monkeys (*Macaca mulatta*, *Macaca fascicularis*).

Additional information is also provided on efferents from the entorhinal and perirhinal cortices. While it is known that the entorhinal cortex has light projections to the retrosplenial cortex (Morris et al.,1999a; Kobayashi and Amaral,^2003^) the termination patterns of these entorhinal efferents remain to be described. Retrograde transport studies have also shown that the perirhinal cortex (especially area 35) projects to the retrosplenial region (Morris et al.,1999a; Kobayashi and Amaral,^2003^), though an anterograde tracing study with injections centered in area 36 did not observe projections from the perirhinal cortex to either area 29 or 30 (Lavenex et al.,^2002^). In contrast, the parahippocampal cortices (areas TH and TF) provide more substantial inputs to the retrosplenial cortex (Kobayashi and Amaral,^2003^), and these projections have been mapped in an anterograde tracer study (Lavenex et al.,^2002^). This study, therefore, just focussed on those medial temporal lobe projections (hippocampus, subiculum, entorhinal cortex, and perirhinal cortex) that have not previously been detailed in an anterograde tracing study. For the same reason, efferents from the amygdala to the posterior cingulate region were also examined in the light of a recent study that used retrograde tracers to uncover light projections from the basal amygdala nucleus to the retrosplenial region (Buckwalter et al.,^2007^).

## MATERIALS AND METHODS

### General Procedure

#### Injections of anterograde tracer (radioactive H^3^ amino acids) into medial temporal lobe

A total of five rhesus monkeys (*Macaca mulatta*) weighing from 1.7 to 7.0 kg, and 16 cynomolgus monkeys (*Macaca fascicularis)* weighing from 2.7 to 5.7 kg received anterograde tracer injections in one of the three principal regions; the amygdala, the hippocampus, or the rhinal cortices. As part of separate behavioral studies, some monkeys (both rhesus and cynomolgus) had previously received surgical transections of the fornix between 2 and 12 months before the injection of the amino acids (all such cases are labeled either ACyF or ARhF). The surgical procedure, which has been described elsewhere (e.g., Bachevalier et al.,^1985^), involved an approach between the rostral cingulate cortices to reach the fornix, via the corpus callosum. For this reason, the “Results” section details whether there was any cortical damage in these cases. Despite the interval since preparing some of the cases, comparisons with sections drawn or photographed soon after initial processing found no evidence that the signal has declined over time.

In a number of cases, bilateral injections were placed within the medial temporal lobe. Such cases are identified with the prefix R or L according to the hemisphere of the particular injection. The lack of published evidence for crossed projections to the retrosplenial cortex from the temporal lobe justifies their inclusion. This decision was supported by the failure to find any crossed projections to the retrosplenial cortex in those cases with injections in just one hemisphere.

All animals were first sedated with ketamine hydrochloride (10 mg/kg), deeply anesthetized with Nembutal (35 mg/kg), and placed in a stereotaxic apparatus or a head holder. Under aseptic conditions, bone and dural flaps were opened to permit access to the temporal lobe. All but three animals received an injection of an equal-parts mixture of tritiated proline (New England Nuclear, L-[2, 3, 4, 5 H], specific activity 139 Ci/mmole) and leucine (New England Nuclear L-[3, 4, 5 H], specific activity 111 Ci/mmole). These injections were made through a 1 μl Hamilton syringe at a final concentration of 50 μCi/μl. In three cases (ERh1, ERh3, and PRh1), the injectate consisted of an equal parts mixture of leucine, lysine, proline, and an amino acid combination derived from an algal protein hydrolysate (Saunders and Rosene,^1988^) at a concentration of 100 μCi/μl. Following injection of the tracer, the dura and skin were sutured. Prophylactic doses of antibiotics were administered to prevent infection (Bicillin 6,000,000 U), whereas dexamethasone phosphate (0.3 mg/kg) was given immediately after surgery to reduce any cerebral oedema. The analgesic, morphine (1–2mg/kg S.C. every 4 h) was given in some, but not all, cases according to the postoperative status of the animal.

After an interval of 6 or 7 days, the monkeys were sacrificed with a lethal dose of Nembutal (100 mg/kg i.v.) and perfused through the left ventricle with normal saline followed by neutral buffered formalin. In the large majority of cases, the brains were cryoprotected with 30% sucrose solution before being cut in 33 μm coronal sections on a freezing microtome. In two cases (ERh1 and ERh3), the brains were embedded in paraffin and cut in 10 μm coronal sections. Every sixth section was mounted on a glass slide from either phosphate buffer or Perfix, coated with Kodak NTB2 emulsion, and stored at 4°C in the dark. For each animal, there was more than one series of sections, and these individual series of sections were stored for between 6 and 30 weeks before development. For the monkeys with entorhinal cortex injections, the longest series was 12 weeks, but for all the remaining monkeys, there was at least one series that was stored for 20 weeks or more. The sections were then developed in Kodak Dl9, fixed, and counterstained with thionine.

### Amygdala Injections

Single injections of between 0.12 μl and 0.2 μl of the amino-acid mixture (i.e., 6–10 μCi) were made in eight cynomolgus monkeys. In an additional monkey (ACy6), a pair of injections (0.20 μl and 0.30 μl, total 50 μCi) was placed in the amygdala in the same hemisphere. All surgeries used a dorsal approach, and the intention was to locate the injections within different amygdala nuclei. Injection coordinates were derived from skull landmarks revealed on X-rays (Aggleton,^1985^). Six monkeys received unilateral injections (ACy6, ACy10, ACy13, ACy16, ACy17, and ACy18), whereas three received bilateral injections (ACy20, ACy21, and ACy22).

### Hippocampal Injections

Seven cynomolgus monkeys (all “ACy” or “ACyF”) and one rhesus monkey (ARhF24) received amino acid injections in the hippocampal formation. Single injections of from 0.13 μl to 0.20 μl (6.5–10 μCi) were made in four cases (ACy12, ACy14, ACyF15, and ACyF19), whereas two (ACy25 and ACy28) of the remaining three monkeys received multiple injections within the same hemisphere, each case totaling 20.5 μCi. In one further case (ACyF27), injections were placed in both hemispheres. In the left hemisphere, a single injection was centered in the caudal subiculum (ACyF27L, 6 μCi), whereas a pair of injections in the right hemisphere involved the rostral presubiculum and caudal perirhinal cortex, as well as the subiculum (ACyF27R, total 20.5 μCi). The coordinates for the hippocampal injections were determined with the aid of electrophysiological recordings made before the injection (Aggleton et al.,^1986^). A tungsten microelectrode was lowered into the hippocampal region and the various cell layers identified by their resting activity.

### Rhinal Cortex Injections

Three rhesus monkeys received amino acid injections using a direct approach into the entorhinal cortex (ERh1, ERh3, and ARhF23). In one case (ERh3), six injections, each of 20 μCi, were placed throughout much of the entorhinal cortex. A second case (ERh1) received a single injection of 10 μCi in area 28M. In the third case (ARhF23), the fornix had been cut bilaterally before an injection of 7 lCi was centered in the very caudal portion of area 28S. Two other monkeys (one rhesus, one cynomolgus) received single injections in the perirhinal cortex. In PRh1, a single injection (20 μCi) was placed in the mid rostral-caudal level of areas 35 and 36. In case ACy9 the injection was located at the caudal limit of areas 35 and 36 (15 μCi).

### Nomenclature

**FIGURE 1:**
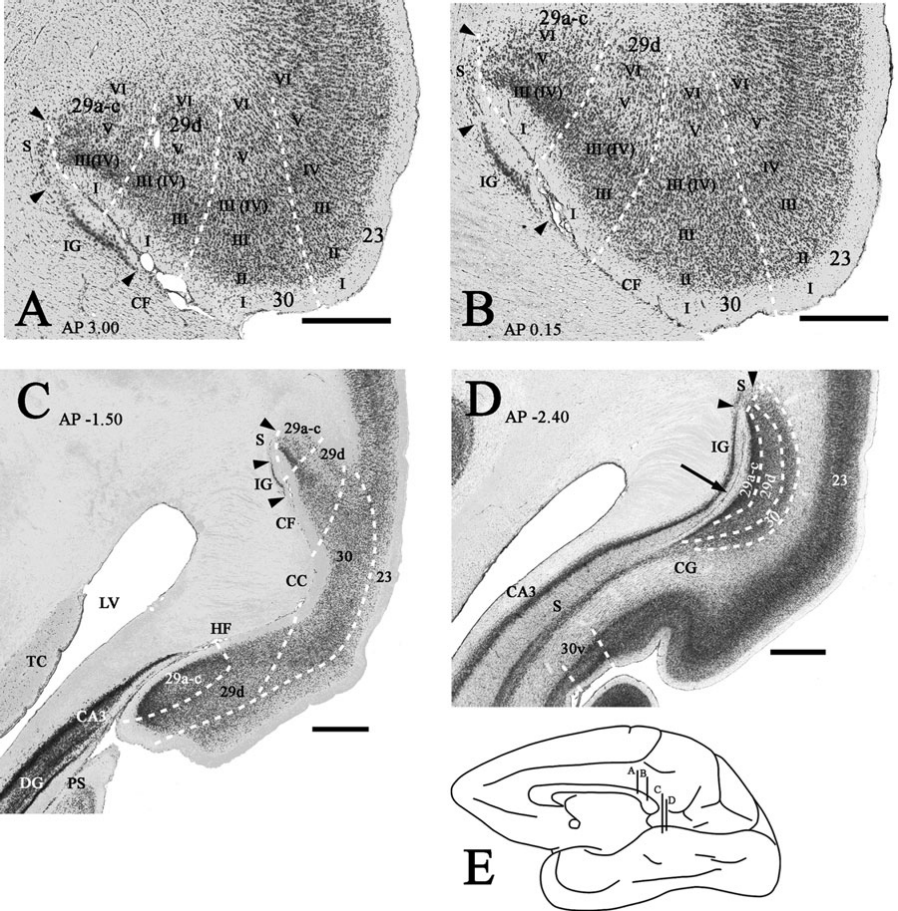
Photomicrographs (A–D) of Nissl stained coronal sections from a cynomolgus macaque (*Macaca fascicularis*) showing the location and arrangement of the retrosplenial cortex (areas 29 and 30). Photomicrographs 1A and 1B, which are the most rostral, show the dorsal retrosplenial cortex, i.e., above the corpus callosum. The numbers indicate the rostro-caudal location of the sections relative to the interaural line (mm) according to the atlas of Paxinos et al. (2009). The outline figure (E), which shows the major features of the medial surface of the macaque brain, has four vertical lines (A–D) that correspond to the positions of the coronal sections in Figures 1A–D. The scale bars on Figures 1A and 1B represent 0.5 mm, the scale bars on Figures 1C and 1D represent 1.0 mm. CC, corpus callosum; CF, callosal fissure; CG, cingulum; DG, dentate gyrus; HF, hippocampal fissure; IG, indusium griseum; LV, lateral ventricle; PS, presubiculum, S, subiculum; TC, tail of caudate. The roman numerals refer to the lamina within the retrosplenial cortex. The arrow on Figure 1D points to the caudal junction of the hippocampal and callosal fissures.

The macaque posterior cingulate region is composed of areas 23, 29, 30, and 31. Of these, areas 29 and 30 comprise the retrosplenial cortex. As the name suggests, much of the retrosplenial cortex is immediately caudal to the splenium of the corpus callosum. That portion above the horizontal level of the corpus callosum will be referred to as the dorsal retrosplenial cortex (Figs. 1A,B) and that portion below the corpus callosum will be referred to as the ventral retrosplenial cortex. Much of the ventral retrosplenial cortex is found within the caudomedial lobule (Kobayashi and Amaral,^2000^). One consequence is that in coronal sections the ventral part of the retrosplenial cortex need not appear continuous with the rest of the (dorsal) retrosplenial cortex.

There are various descriptions of the cytoarchitecture and borders of the retrosplenial cortex in macaque monkeys (Morris et al.,1999a; Kobayashi and Amaral,^2000^; Vogt et al.,^1987^, ^2005^; Vogt,^2009^). All of these descriptions distinguish area 30 as dysgranular, whereas area 29 is granular (Fig. 1). The term granular for area 29 does not, however, refer to a granular layer IV (as would be the case in isocortex), rather the term refers to the densely packed granular looking neurons that occur immediately deep to the molecular layer I. This “granular” cell layer, which is sometimes referred to as layer III(IV) (Morris et al.,1999a; Vogt et al.,^2005^; Vogt,^2009^), is a distinctive feature of much of the retrosplenial cortex.

Area 29 can be split into two subdivisions (Fig. 1), and these subdivisions have been given different names by different authors. This study employs the terminology (29a–c and 29d) as used by multiple authorities (e.g., Vogt et al.,^1987^; Morris et al.,1999a; Shibata and Yukie, 2003; Paxinos et al.,^2009^). An additional reason for using this particular terminology is that the various subregions have been described in the coronal plane (Morris et al.,1999a; Paxinos et al.,^2009^), and so can be aligned with the present material (also coronal). Even so, it is important to appreciate that the three-dimensional shape of the retrosplenial cortex means that not all cytoarchitectonic distinctions can be made with confidence from coronal sections (see also Morris et al.,1999a; Kobayashi and Amaral,^2000^). As the amino acid injections were made in order to examine a wide variety of projection sites (Aggleton et al.,^1986^; Friedman et al.,^2002^; Aggleton et al.,^2005^), it was not possible to modify the plane of section for this particular study.

The subdivision of area 29 furthest from the midline is designated area 29a–c (Vogt et al.,^1987^; Morris et al.,1999a; Paxinos et al.,^2009^). Area 29a–c is buried deep in the callosal fissure (Fig. 1). The same (lateral) subdivision is also called area 29l by other authorities (Kobayashi and Amaral,^2000^; Vogt et al.,^2005^). The subdivision of area 29 closest to the midline is area 29d (Vogt et al.,^1987^; Morris et al.,1999a; Paxinos et al.,^2009^). This more medial subdivision (29d) has also been called 29m (Kobayashi and Amaral,^2000^; Vogt et al.,^2005^). The medial border of area 29d (29m) is with area 30 (Fig. 1).

In addition to the alternate terminologies for the major subdivisions within area 29, there are also differences in how the various lamina are designated. In this study (Fig. 1), we use the terms layer I (molecular) and layers III(IV), V, VI for the cellular lamina in 29a–c (Morris et al.,1999a; Vogt et al.,^2005^). In contrast, Kobayashi and Amaral (2000) use the terms external pyramidal, internal pyramidal, and polymorphic for the three cellular layers in area 29a–c (their 29l). The border of 29a–c with 29d is most clearly marked by the appearance of an additional layer III (Vogt and Pandya,^1987^; Morris et al.,1999a) making four cell layers in 29d (Fig. 1). Neither 29a–c nor 29d have a layer II (Fig. 1). It should be noted that Kobayashi and Amaral (2000) describe only three cell layers in both 29a–c and 29d as the “additional” layer III in 29d (29m) is thought to comprise interposing cells from layers II and III of the adjacent area 30. This study adheres to the descriptions of Morris et al. (1999a) and Paxinos et al. (2009), i.e., four cell layers in area 29d (Fig. 1). Area 30 (“dysgranular”) is distinguished by the gradual loss of the “granular” layer III(IV) that is so distinctive in area 29, along with the beginnings of a layer II and the greater development of pyramidal layers III and V (Fig. 1).

Medial to area 30 is area 23. Although their border is not precise, area 23 has a more distinct layer IV and often shows lamination within layer V. As a consequence, area 23 has the six laminar subdivisions found in isocortex. Despite agreements on these principal cytoarchitectonic features, there remain some differences between authorities over the border between areas 23 and 30 (see Kobayashi and Amaral,^2000^; Vogt et al.,^2005^). Again, this study uses the landmarks described by Morris et al. (1999a; see also Paxinos et al.,^2009^). The one exception is that, following the practice of Kobayashi and Amaral (2000), we distinguish a specific ventral area 30 (Fig. 1D). This area 30V is a transitional zone located in the dorsolateral bank of most rostral calcarine sulcus and occupies part of the rostral prostriate cortex as demarcated by Paxinos et al. (2009).

Two final points relate to the complex three-dimensional shape of the retrosplenial cortex as it wraps around the back of the splenium. The first is that immediately caudal to the splenium, the hippocampal fissure and the callosal fissure extend dorsally and ventrally, respectively, to join together and fuse. Their junction creates a distinctive crescent shaped fissure that is lined by the subiculum and indusium griseum on its lateral side and by the retrosplenial cortex (area 29) on its medial side (Figs. 1C,D). This junction is repeatedly referred to in the text. The second point is that the border between the presubiculum and the retrosplenial cortex in the caudomedial lobule can appear very subtle (Kobayashi and Amaral,^2000^), and when using a Nissl stain, it is sometimes not possible to be definitive about this transition.

The designation of the amygdala nuclei follows that described by Friedman et al. (2002) and Amaral et al. (1992), whereas that of the various hippocampal subfields and regions follows Lorente de Nó, (1934) [see also Paxinos et al. (2009)]. The entorhinal divisions are taken from Saunders and Rosene (1988).

### Data Analysis

The sections were examined under both bright-field and dark-field conditions using Leica Laborlux and Leica DM microscopes. One concern with autoradiography is that it can be difficult to distinguish fibers from termination. In this study, elongated threads of silver grains were assumed to be fibers, whereas dust-like aggregations of grains concentrated in particular laminar were assumed to reflect termination. Layer I (the molecular layer) of the retrosplenial cortex did, however, pose particular difficulties as it consistently contained fibers as well as separate grains, the latter suggesting termination. Given that this layer may contain fibers running at right angles to the plane of section, which could appear as separate grains, caution was used when trying to distinguish the nature of any label in layer I. In a number of cases, there was a clear reduction of label in layer I of area 29 when progressing through consecutive sections away from the injection site. At the same time, there was no evidence of other label in adjacent cingulate areas. This pattern of label was taken as evidence of termination, but it could not be definitive.

## RESULTS

The monkeys used in this study were from two closely related species. To help distinguish the species, all cynomolgus monkeys have “Cy” in their designation, whereas all rhesus monkeys have “Rh” in their designations. Those monkeys with a fornix transection have the additional letter F in their designation.

### Amygdala Projections

**FIGURE 2:**
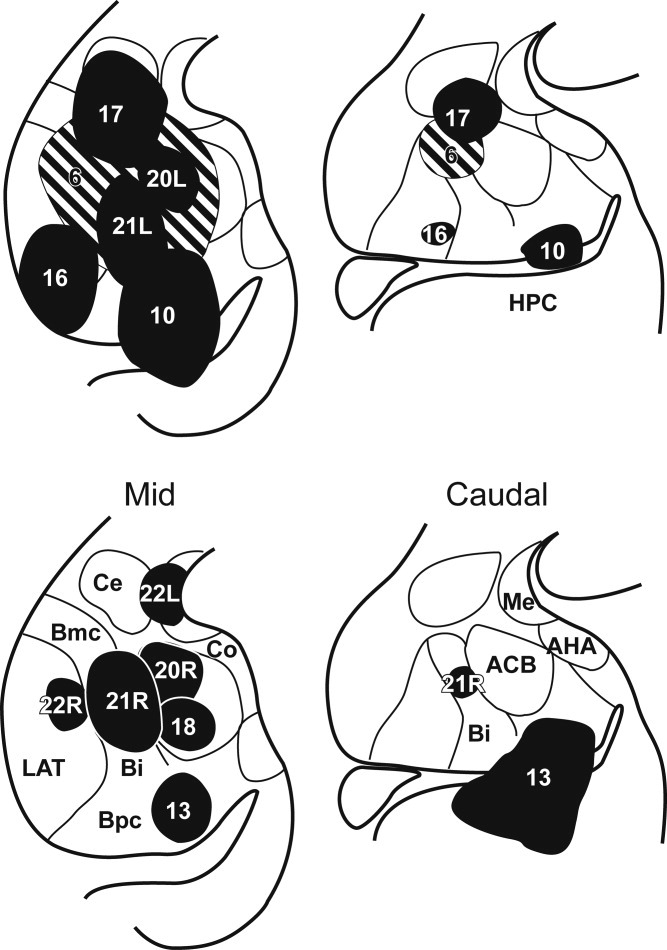
Location and extent of the amino acid injections into the amygdala. The injection sites are depicted on standard coronal sections at the level of the middle and caudal third of the amygdala. ACB, accessory basal nucleus; AHA, amygdalo-hippocampal area; Bi, basal nucleus, intermediate division; Bmc, basal nucleus, magnocellular division; Bpc, basal nucleus, parvocellular division; Ce, central nucleus; Co, cortical nucleus; HPC, hippocampus; LAT, lateral nucleus; Me, medial nucleus.

Figure 2 depicts the placement and extent of the amino acid injections into the amygdala. Individual injection areas are considered to correspond to the region in which silver grains filled the neuropil and perikarya at a density that was appreciably above background. Each of the major nuclei, i.e., the basal, lateral, accessory basal, central and medial nuclei, was injected (Fig. 2). In none of the cases with an injection site confined to the amygdala was there evidence of a projection that terminated within the retrosplenial cortex or area 23.

**FIGURE 3:**
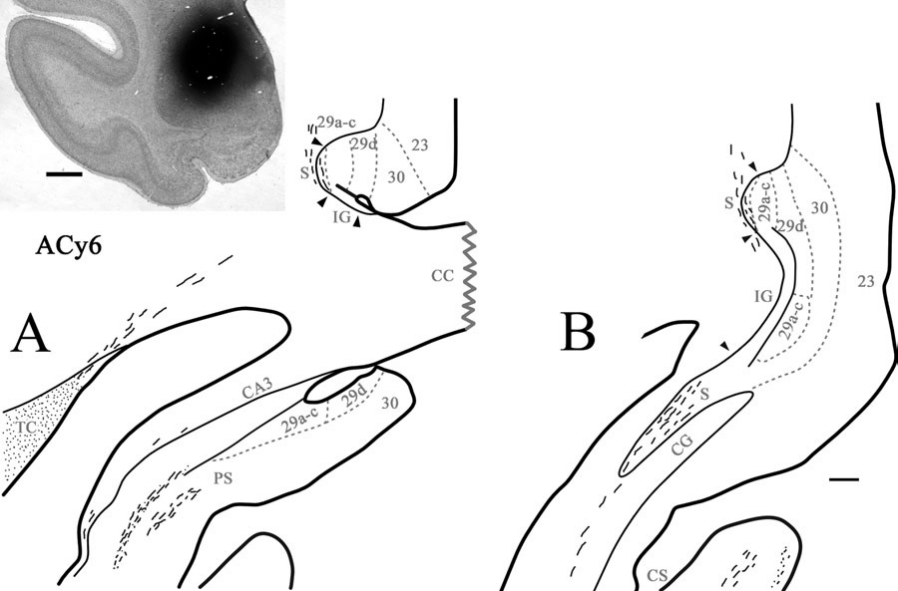
Drawings of two coronal sections showing the path of autoradiographic label in the posterior cingulate region in the case with the largest amygdala injection (ACy6). (Fig. 3A is the more rostral.) While labeled fibers were present in the posterior cingulate region, no termination could be seen. CC, corpus callosum; CG, parahippocampal cingulum; CS, calcarine sulcus; IG, indusium griseum; LV, lateral ventricle; PS, presubiculum; S, supracallosal subiculum; TC, tail of caudate. The scale bar on the photomicrograph represents 2.0 mm, whereas that on the drawing represents 500 μm.

Those injections involving the basal nuclei are of particular interest as there is evidence from retrograde tracers (Buckwalter et al.,^2007^) that these nuclei project lightly to the retrosplenial cortex. Attention, therefore, first focussed on case ACy6, which had a large dual injection centered on the basal nucleus (magnocellular and intermediate) (Figs. 2 and 3). Both labeled fibers and terminal label were present in the subiculum at its most caudal level, with fibers running in a dorsomedial direction through the presubiculum toward the caudomedial lobule of the retrosplenial cortex. There was, however, no evidence of any termination in the retrosplenial cortex (Fig. 3). More caudally, a few labeled fibers could be seen running dorsally in the white matter lateral to area 23 behind the splenium. A small number of labeled fibers were also found in the supracallosal subiculum immediately lateral to area 29a–c (Fig. 3), but again no retrosplenial termination could be discerned.

This pattern was repeated in a case with more discrete injections centered in the basal nucleus in both the left and right hemispheres (Fig. 2; ACy21L,R). Labeled fibers again emerged dorsally through the presubiculum at the caudal limit of the hippocampus, and a few fibers could be seen in the supracallosal subiculum, but there was no convincing evidence of terminal label in areas 29 or 30 in either hemisphere. Likewise, an injection centered in the parvocellular basal nucleus (ACy10) produced a few labeled fibers running dorsally from the most caudal subiculum into the retrosplenial region, but no sign of termination.

The only exception to this pattern of no retrosplenial label after an amygdala injection was case ACy13, but here the injection spread from the medial basal nucleus to involve the genu of the hippocampus (Fig. 2). Now, labeled fibers were not only present in the most caudal subiculum and presubiculum, but there was also evidence of light label in the molecular layer of a very restricted portion of ventral area 29 in the caudomedial lobule, i.e., below the corpus callosum. This label, presumably originating from the genu of the rostral hippocampus, was found in layer I of 29a–c (on the lateral edge of the caudomedial lobule) and in layer I of 29d (on the medial surface of the caudomedial lobule). As described below, this pattern matches that found for rostral subicular injections.

### Hippocampal (Subicular) Projections

**FIGURE 4:**
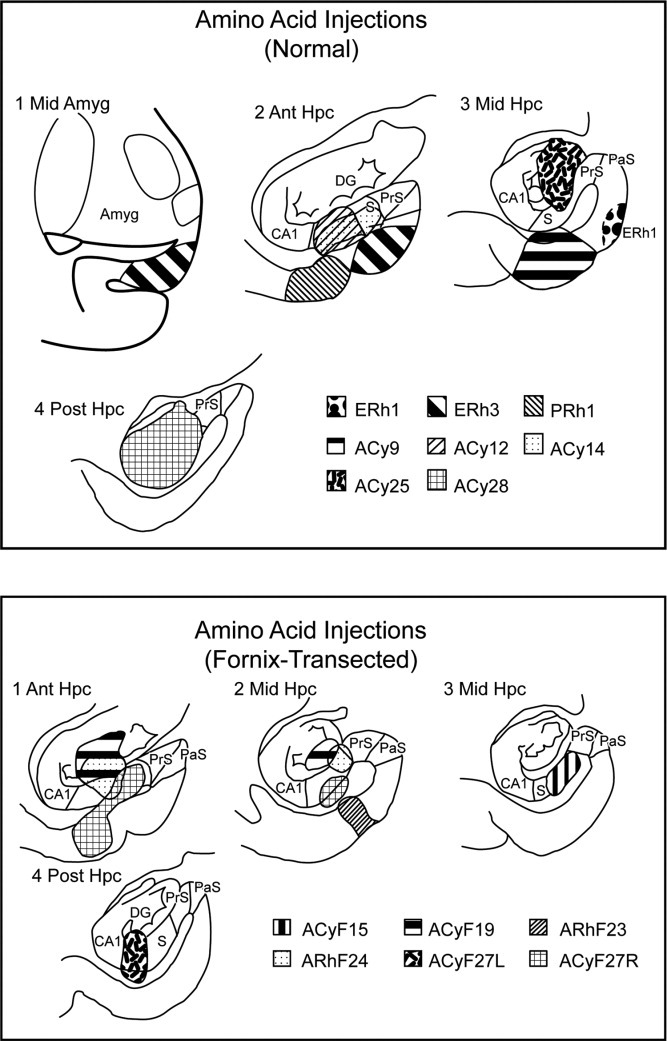
Location and extent of the amino acid injection placements in the hippocampal formation drawn onto standard coronal sections. The injection sites in the normal animals are depicted in the upper box, those in cases where the fornix had been transected before injection are shown in the lower box. Amy, amygdala; DG, dentate gyrus; Hpc, hippocampus; PaS, parasubiculum; PrS, presubiculum; S, subiculum.

All but one of the cases with hippocampal formation injections had anterogradely transported label in the retrosplenial cortex. In the sole exception (ACyF19), the injection was centered in the rostral half of the dentate gyrus and did not involve CA1, the subiculum, or presubiculum (Fig. 4). No retrosplenial label was found in this case. Comparisons across the other cases showed that while projections from the rostral subiculum (including the genu) terminate in more ventral retrosplenial sites, e.g., in the caudomedial lobule, the caudal subiculum provides denser projections that additionally terminate in caudal and dorsal retrosplenial sites. Careful comparisons between those cases with and without fornix transection found no evidence to suppose that transection of the fornix affected the transport of label to the retrosplenial region.

#### Rostral subiculum

**FIGURE 5:**
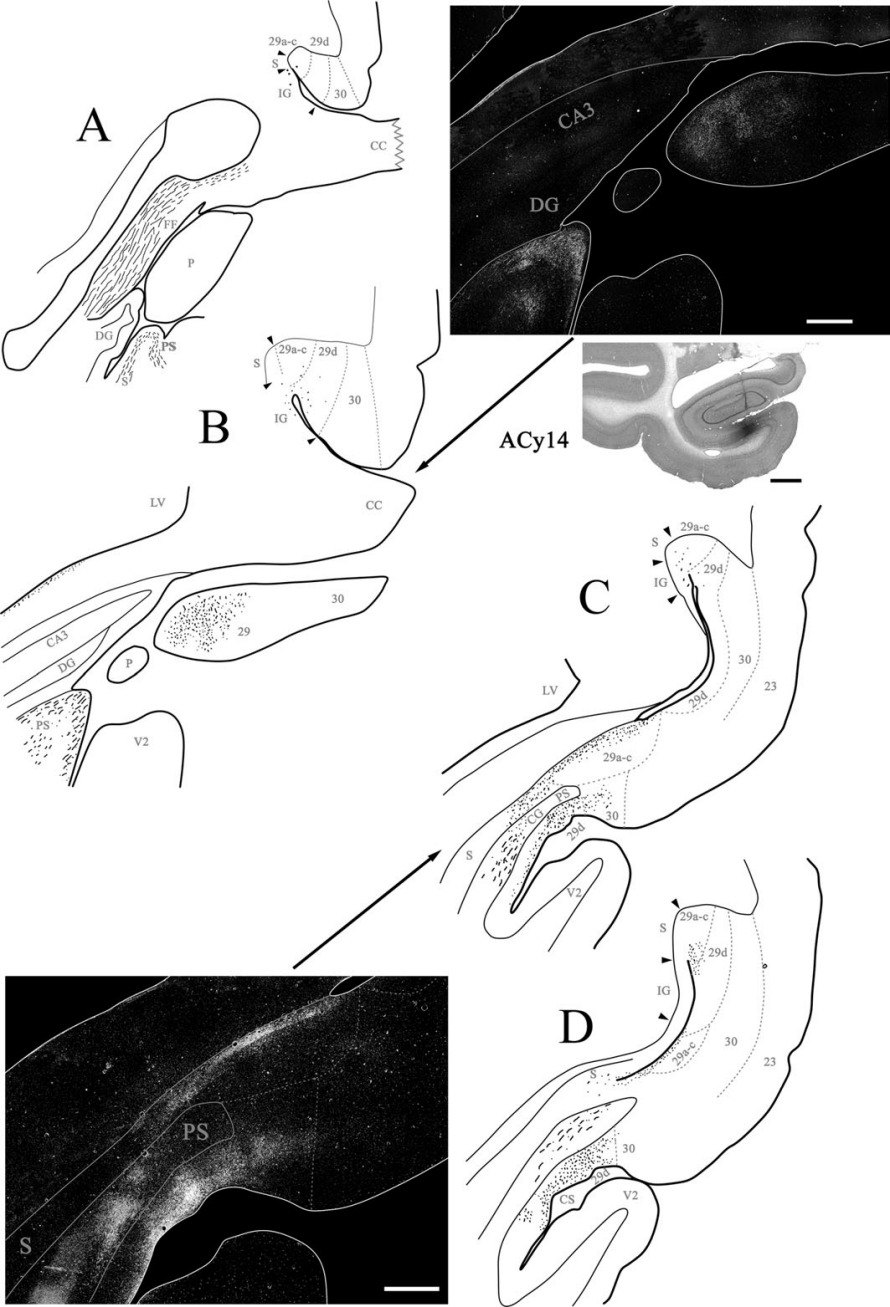
Case ACy14—rostral subiculum injection. Dark-field photomicrographs and line drawings of coronal sections through the posterior cingulate region showing the distribution of anterograde label, including fibers, after an amino acid injection centered in the rostral subiculum. Sections A–D go from rostral to caudal. The locations of the dark-field photomicrographs are indicated by arrows. CC, corpus callosum; CG, parahippocampal cingulum; DG, dentate gyrus; FF, fimbria fornix; IG, indusium griseum; LV, lateral ventricle; P, pulvinar; PS, presubiculum; S, subiculum; TC, tail of caudate. The scale bar on the bright-field photomicrograph represents 2.0 mm, whereas that on the dark-fields represents 500 μm.

In two cases, single injections were centered in the rostral subiculum (ACy12 and ACy14). In a third case, the injection spread across from the dentate gyrus into the adjacent subiculum (ARhF24) at the anterior level of the hippocampus (Fig. 4). The injection in ACy12 was a little more proximal than that in ACy14 and so reached the border with CA1, whereas the injection in ACy14 (Figs. 4 and 5) was more distal and so reached the border with the presubiculum. The main features of the transported label in the posterior cingulate region were very similar for all three cases, with labeled fibers reaching the retrosplenial cortex and evidence of termination in the caudomedial lobule, i.e., below the corpus callosum. Little label was seen in the dorsal retrosplenial cortex.

Figure 5 shows four coronal sections from case ACy14, which is representative of these three cases. The subiculum injection in this case extended ∼1.5 mm in the AP plane. At the caudal limit of the hippocampus (Fig. 5C), labeled fibers ran through the molecular (plexiform) layer in the most dorsolateral part of the subiculum to reach layer I of ventral area 29a–c, i.e., in the caudomedial lobule, where additional label was present in layer III (IV) of the same area (Figs. 5B,C). Other fibers ran through the cingulum and presubiculum where they also reached area 29 in the caudomedial lobule. Some of these fibers then terminated in the caudal retrosplenial cortex (layers I and III) within a discrete area of the lateral bank of the calcarine sulcus (Figs. 5C,D). Most of this area has features consistent with area 29d, although this same patch of label continued caudally into 30V (Kobayashi and Amaral,^2000^), i.e., within the prostriate area as designated by Paxinos et al. (2009). Within the caudomedial lobule, some label spread dorsally in the molecular layer of area 29 just medial to the junction of the callosal and hippocampal fissures, resulting in a restricted patch of label behind the splenium that was found predominantly in layer I of 29a–c at the very caudal limit of the fissures (Fig. 5D). At this very caudal level, light label extended into layer III (IV) of 29a–c and into the immediately adjacent parts of layers I and III(IV) of 29d. The silver grains in layer III(IV) resembled terminal label. In the more rostral dorsal retrosplenial cortex only very light label was occasionally seen in layer I of dorsal 29a–c. Much of the label in layer I of 29a–c did not appear as fibers, and it assumed that some of this label reflected termination in this layer.

The pattern of label in case ACy12 was very similar to that just described for ACy14. Again, a line of continuous label could be traced in ACy12 from the caudal subiculum to layer I and some of layer III of area 29 in the ventral caudomedial lobule. Although some of this label comprised fibers there appeared to be a clear projection to the lateral and ventral portions of the caudomedial lobule (area 29). Only occasional label was seen in layer I of area 29a–c dorsal to the corpus callosum. Unlike ACy14, there was no label in layer III(IV) of area 29 at the caudal level of the junction of the hippocampal and callosal fissures.

The retrosplenial label in Case ARhF24 (injection in rostral dentate gyrus and subiculum) was very similar to that seen in the other two cases. The main feature was the clear evidence of both fibers and termination within areas 29a–c and 29d in the caudomedial lobule. This label was heaviest in layer I but also extended into layer III(IV). Most of the fibers reaching this part of 29 appeared to pass through the molecular (plexiform) layer of the subiculum, with lighter projections running though the cingulum and then presubiculum. At the most caudal level of the retrosplenial cortex there was a clear, but light band of continuous label in the molecular layer of areas 29a–c and 29d where the hippocampal and commissural fissures meet. From the appearance of the label it is again supposed that some of the silver grain in the molecular layer reflected termination. Some light label was also found in the adjacent layer III(IV) of just area 29a–c (like ACy14). Unlike ACy12 and ACy14, there was no evidence of a projection to the bank of the calcarine sulcus. Finally, it should be noted that fornix surgery did not appear to result in any damage to the cingulate cortices.

#### Mid levels of the subiculum

**FIGURE 6:**
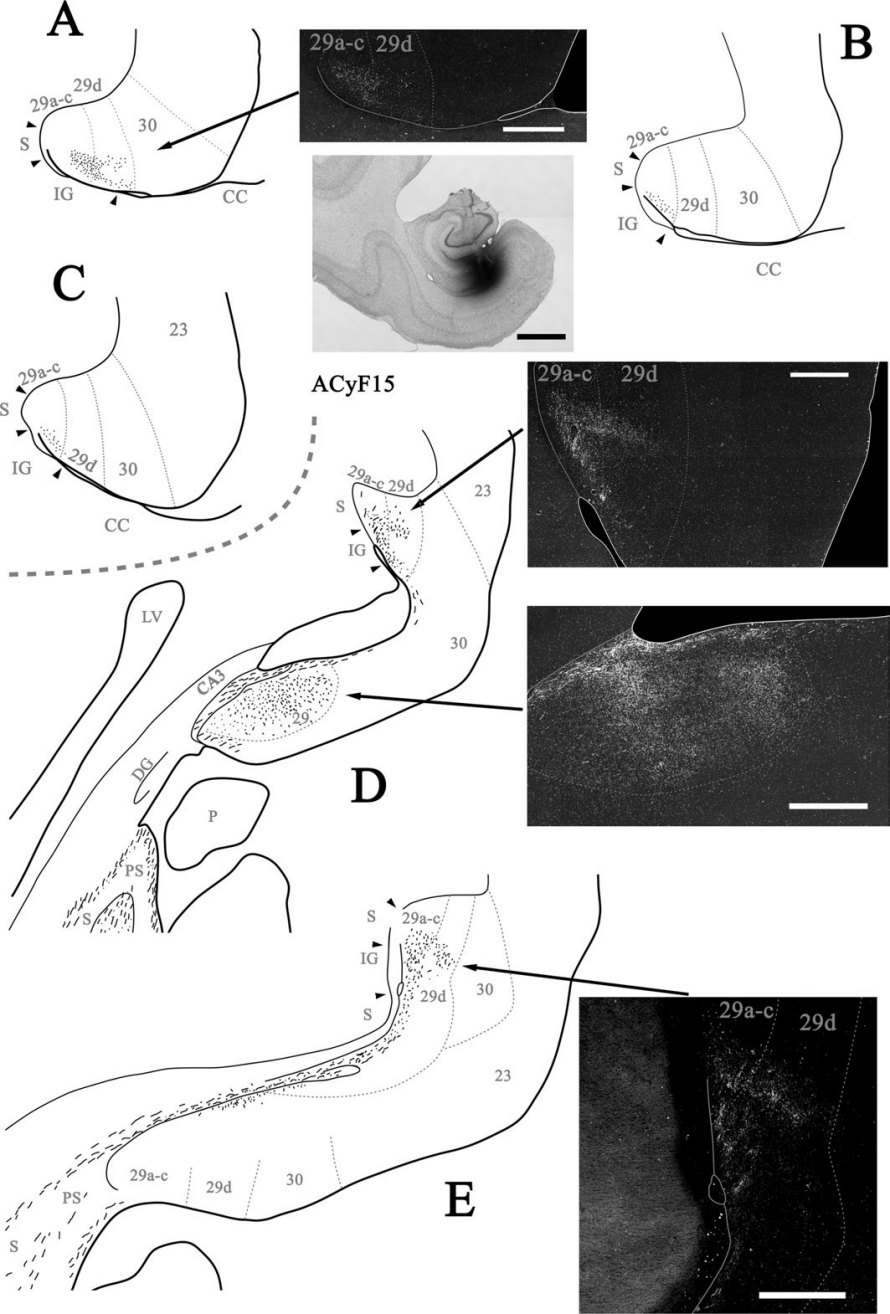
Case ACyF15—mid subiculum injection. Dark-field photomicrographs and line drawings of coronal sections through the posterior cingulate region showing the distribution of anterograde label, including fibers, after an amino acid injection centered in the mid subiculum. Sections A–E go from rostral to caudal. The locations of the dark-field photomicrographs are indicated by arrows. CC, corpus callosum; DG, dentate gyrus; IG, indusium griseum; LV, lateral ventricle; P, pulvinar; PS, presubiculum; S, subiculum. The scale bar by the bright-field photomicrograph represents 2.0 mm, whereas that on the dark-fields represents 500 μm.

In two cases (ACyF15 and ACy25), the injections involved the mid AP levels of the subiculum. In case ACy25, the injection was centered in the hippocampus proper but spread into the adjacent subiculum (Fig. 4). In case ACyF15, the injection was centered within the subiculum but may have involved some of the deepest cells of the presubiculum at its border with the subiculum (Figs. 4 and 6). The injection in case ACyF15 extended ∼1.5 mm in the AP plane. While both cases had similar patterns of retrosplenial label, transported label was more evident in ACyF15, presumably reflecting the greater subicular involvement.

In contrast to the rostral subicular injections described above, case ACyF15 contained appreciable label in dorsal area 29 (Fig. 6A). Labeled fibers left the most caudal parts of the hippocampus to enter or pass through the ventral retrosplenial cortex. One set of fibers passed through the most caudal part of the presubiculum to terminate in the adjacent caudomedial lobule, with appreciable label in both layer III(IV) and layer I of area 29a–c (Fig. 6D). The majority of labeled fibers passed via the molecular layer of the subiculum and then wrapped around the lateral edge of area 29 where it adjoins the merging of the callosal and hippocampal fissures behind the splenium (Figs. 6D,E). As these labeled fibers passed through layer I of 29a–c and 29d en route to the dorsal retrosplenial cortex, some termination may have occurred. Additional termination was found in more dorsal area 29 (Figs. 6A–C). Cadval to the splenium, label was evident in both layer I and layer III(IV) of 29a–c and of 29d (Fig. 6D), with the densest label in 29a–c. Immediately rostral of the splenium this dorsal retrosplenial label became restricted to layer I of 29a–c (Figs. 6B,C), but moving more anterior (above the level of the habenula) light label was again present in layer I and layer III(IV) of area 29a–c (Fig. 6A). Some of this label extended into 29d [layer I and layer III(IV)]. Once again, it is supposed that some of the layer I label reflected termination. Finally, it should be added that the fornix surgery in this case resulted in some bilateral superficial tissue damage in rostral area 30 but this was confined to the most rostral limit of this area, i.e., at the transition to area 24.

#### Caudal hippocampus

**FIGURE 7:**
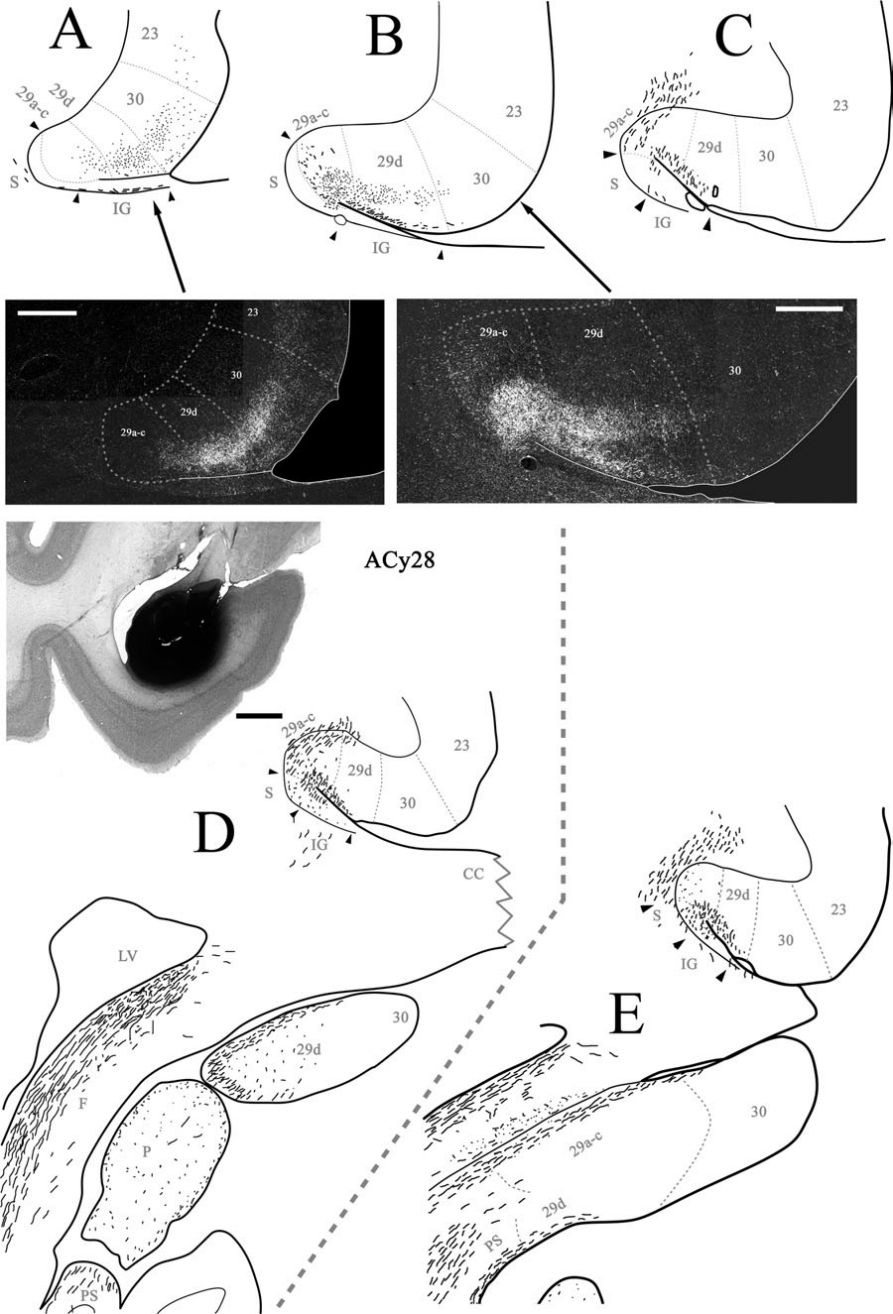
Case ACy28—caudal hippocampus injection (see also Fig. 8). Dark-field photomicrographs and line drawings of coronal sections through the posterior cingulate region that show the distribution of anterograde label, including fibers, after an amino acid injection centered in the caudal hippocampus, including the subiculum. Sections A–G go from rostral to caudal. The locations of the dark-field photomicrographs are indicated by arrows. CC, corpus callosum; FF, fimbria fornix; IG, indusium griseum; LV, lateral ventricle; P, pulvinar; PS, presubiculum; S, subiculum. The scale bar on the bright-field photomicrograph represents 2.0 mm, whereas that on the dark-fields represents 500 μm.

**FIGURE 8:**
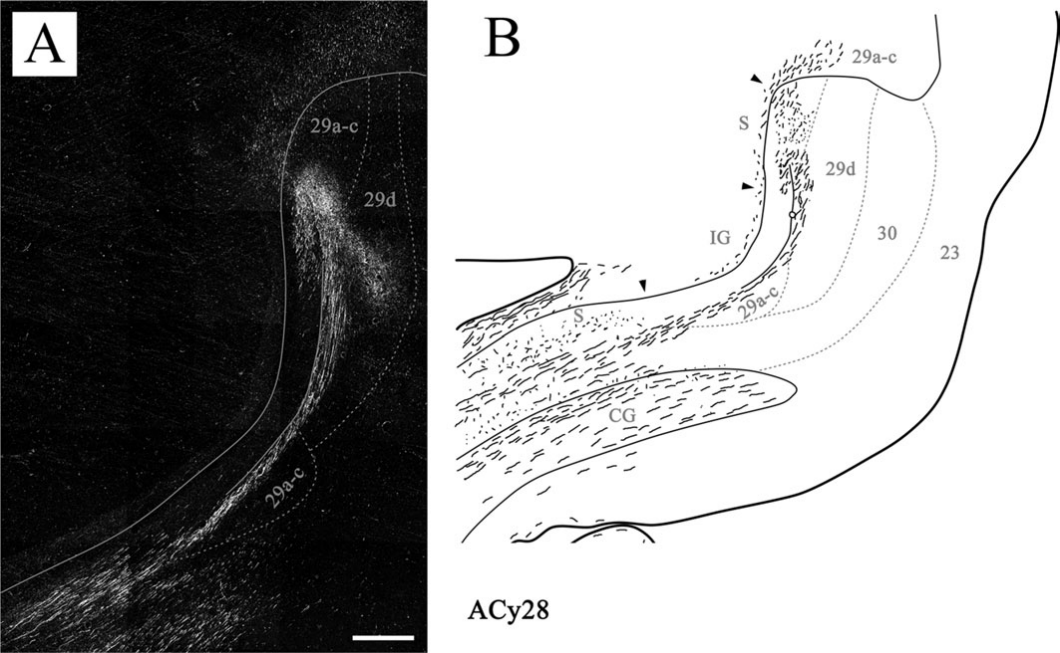
Case ACy28 (continued from Fig. 7)—caudal hippocampus injection. Figures 8A and 8B depicts two sections just caudal to those in Figure 7E. The coronal section in the dark-field photomicrograph (8A) is just anterior to line drawing of a coronal section at the posterior limit of the retrosplenial cortex (8B). Both sections 8A and 8B show the distribution of anterograde label, including fibers, after an amino acid injection centered in the caudal hippocampus, including the subiculum. CG, parahippocampal cingulum; IG, indusium griseum; S, subiculum. The scale bar represents 500 μm.

Two cases (ACyF27l and ACy28) received injections in the caudal hippocampus that involved the subiculum, although the extent of these injections was very different (Fig. 4). Case ACy28 had one of the largest hippocampal injections (Figs. 4 and 7) as it appeared to fill the entire caudal hippocampus, involving the dentate gyrus, the CA1–4 fields, the subiculum, and adjacent parts of the presubiculum. This large injection appeared spherical and so extended almost 5.0 mm in the AP plane. A resulting dense band of labeled fibers passed through the molecular layer of the very caudal subiculum and presubiculum to run in layer I of area 29, immediately medial to the junctions of the callosal and hippocampal fissures, and so continue in a caudal and dorsal direction toward the splenium (Figs. 7D,E and 8A). A second, lighter band of fiberspassed through the temporal cingulum and presubiculum to run through the molecular layer of area 29d on the medial surface of the caudomedial lobule (Figs. 7E and 8B). It is possible that there was some light termination in layer III(IV) of 29a–c in the ventral retrosplenial cortex in the caudomedial lobule.

Starting from the most caudal part of the dorsal retrosplenial cortex (Fig. 8B), a dense zone of labeled fibers could be followed rostrally in layer I of 29a–c, along with additional fibers in layer I of 29d (Figs. 8A,B). A patch of terminal label was also present in layer III(IV) of 29a–c and 29d near the caudal limit of the retrosplenial cortex (Fig. 8A). From a little way in front of the splenium (Fig. 7B) to the very rostral limit of the dorsal retrosplenial cortex (Fig. 7A), a clear band of termination reappeared across layer III(IV) of both 29a–c and 29d. This label became denser going rostrally and also shifted medially, so that more of the label in layer III(IV) was found in 29d, and some of this label extended into adjacent area 30 [layer III(IV)]. Above the level of thalamic nucleus medialis dorsalis, this label in III(IV) extended completely across area 30 to reach adjacent parts of area 23 (deep layer III) (Fig. 7A). Labeled fibers were also present in layer I across area 29 and, to a lesser extent, in area 30. The terminal label in layer III(IV) of areas 29 and 30 stopped near the rostral limit of the retrosplenial cortex, though a small number of fibers continued to progress in the molecular layer of 29a–c just above the corpus callosum to the level of the anterior thalamic nuclei. This label, which appeared to be predominantly fibers, could be followed in the molecular layer of the cingulate cortex immediately dorsal to the indusium griseum as far forward as the genu of the corpus callosum. In addition, *en passage* labeled fibers could often be seen running dorsally through the surpracallosal subiculum and adjacent dorsal area 29a–c (Figs. 7C,D,E and Fig. 8).

In case ACyF27L the injection was appreciably smaller in extent than ACy28 and was predominantly in CA1 and the neighboring (proximal) part of subiculum, i.e., in the prosubiculum (Lorente de Nó,^1934^). Labeled fibers passed dorsally through the molecular layer of the caudal subiculum to continue medial to the junction of the hippocampal and callosal fissures in molecular 29a–c, and then accumulate in layer I of dorsal 29a–c. Rostral to the splenium light label was present in layer I of 29a–c, though it sometimes extended medially into 29d. No label could be seen in either the supracallosal subiculum or the indusium griseum. The light label in layer I of area 29 extended as far forward as the posterior portion of nucleus medialis dorsalis. No label could be seen in any other retrosplenial areas, suggesting that the layer I label included terminal fibers. The fornix surgery in this case completely spared the cingulate cortices in both hemispheres.

### Rhinal Cortex Projections

Cases with injections in the entorhinal and perirhinal cortices were characterized by labeled fibers that predominantly passed lateral (not medial) to the junction of the callosal and hippocampal fissures. While label was consistently found in the indusium griseum and supracallosal subiculum, only very light label was present in the molecular layer of dorsal 29, and only after entorhinal injections.

#### Entorhinal

**FIGURE 9:**
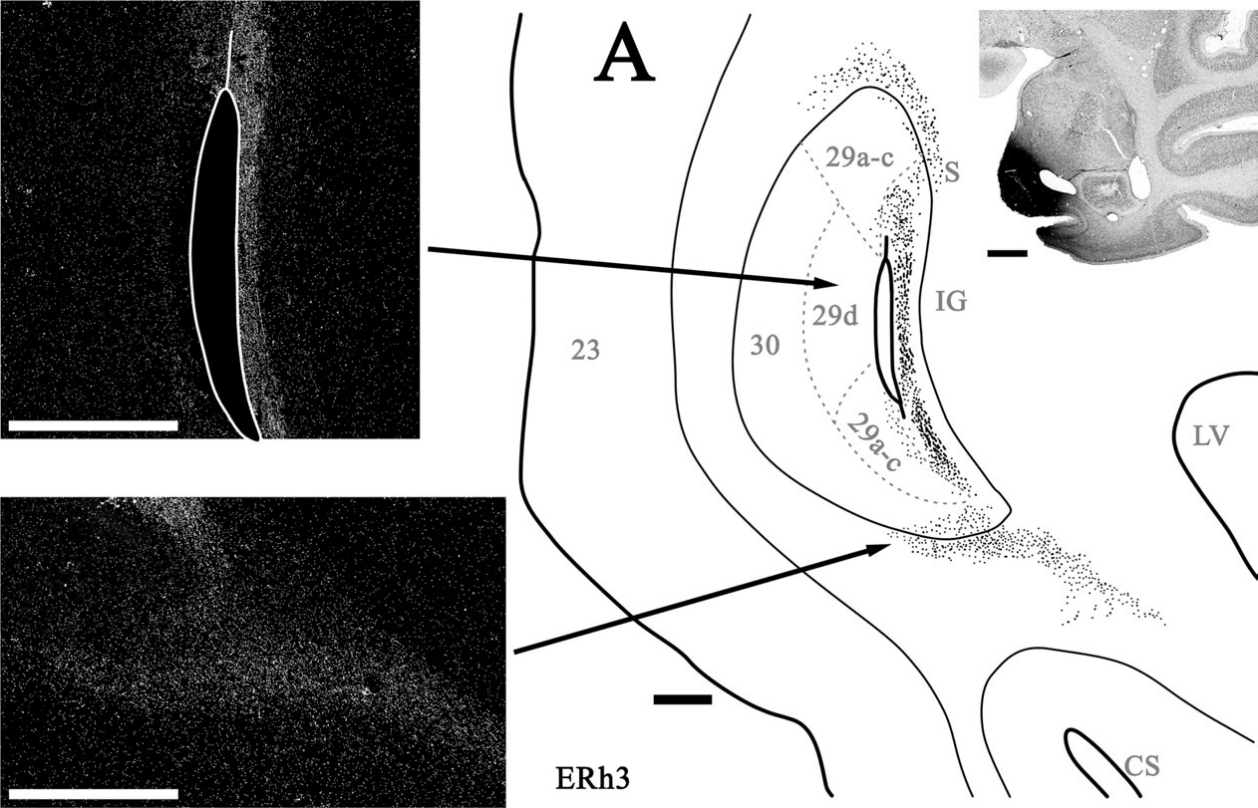
Case ERh3—entorhinal cortex injection. Dark-field photomicrographs and line drawings of a coronal section at the caudal limit of the retrosplenial cortex, showing the distribution of anterograde label, including fibers. The locations of the dark-field photomicrographs are indicated by arrows. CS, calcarine sulcus; IG, indusium griseum; LV, lateral ventricle; S, subiculum. The scale bar by the bright-field photomicrograph represents 2.0 mm, whereas that on the dark-field photomicrographs represents 500 μm.

Of the three cases with entorhinal injections, ERh3 is described first as it received the largest injection (Fig. 4). Labeled fibers traversed the most caudal subiculum, presubiculum, and CA3 in a dorsal direction, so that much label appeared in the molecular layers of the subiculum and indusium griseum just lateral to the junction of the callosal and hippocampal fissures (Fig. 9). Very light label was also present in the molecular layer of dorsal 29a–c at its most caudal limit. Adjacent to the label in the dorsal subiculum was an extensive patch of fibers in the white matter immediately lateral and dorsal to 29a–c, with some fibers heading toward to the dorsal cingulum (Fig. 9). Going rostral toward the splenium, light label continued in the molecular layer of 29a–c, seemingly coming from the adjacent supracallosal subiculum. Further rostral, the light label in layer I of 29a–c continued but the label in the supracallosal subiculum and the indusium griseum, as well as that in the supracallosal fornix, was far more consistent. On just a couple of sections the layer I label extended medially into areas 30 and 23, but then disappeared on adjacent sections (see also ARhF23). In ERh3 a separate, light projection to the ventral retrosplenial cortex could also be seen. Fibers passing through layer I of the most caudal presubiculum continued in a dorsomedial direction into the caudomedial lobule. Light label was present in layers I and II of area 30 just below the splenium. Finally, an additional light projection was seen in layers I and II of area 30V, in the calcarine sulcus.

The injection in case ERh1 was the smallest of the three within the entorhinal cortex and was centered in 28M (Fig. 4). The pattern of projections to dorsal 29a–c was consistent with the previous entorhinal case but more restricted, and the only label in the ventral retrosplenial cortex was found in 30V (layer I) in the prostriate cortex. As in ERh3, the large majority of fibers passed lateral to the callosal/hippocampal fissure junction (i.e., in the molecular layer of the subiculum), with just a little label more medial in the molecular layers of areas 29 and 30.

In the third case (ARhF23), the injection was placed at the caudal limit of 28S and reached the border with area 35 and may have involved the immediately adjacent subiculum. The route of the labeled fibers matched that described in ERh1 and ERh3, and again there was clear label in the dorsal subiculum and indusium griseum. Like ERh1 there was some restricted, light label in layer I of areas 29a–c and 29d in the rostral portion of the dorsal retrosplenial cortex. At this same level, there was also light label in layer III of 29d.

#### Perirhinal

In two cases an injection was centered in the perirhinal cortex, where it predominantly involved area 36, but also included area 35 (PRh1 and ACy9). In both cases, labeled fibers were seen immediately adjacent to the retrosplenial cortex, but there was no clear evidence of termination within areas 29 or 30. In both cases, labeled fibers emerged dorsally from the molecular layer of the most caudal subiculum, presubiculum, and CA3 to pass lateral to the merging of the callosal and hippocampal fissures at the most caudal limits of the retrosplenial cortex. This label reached the indusium griseum, but more completely filled the molecular layer of the supracallosal subiculum. At this caudal level, *en passage* fibers were also apparent just lateral and dorsal to 29a–c but no label was present within the retrosplenial cortex itself. At levels just rostral to the splenium, labeled fibers in case PRh1 could be traced in the lower bank of the callosal fissure, so that label filled up the molecular layers of the supracallosal subiculum, the indusium griseum, and, more medially, parts of the supracallosal fornix. Some of the label in the supracallosal subiculum of PRh1 appeared to continue through to the area of labeled fibers in the white matter immediately lateral and dorsal to area 29a–c (above the retrosplenial cortex). In just case PRh1, extremely light label was sometimes seen in that part of layer I of lateral 29a–c immediately adjacent to the supracallosal subiculum. Continuing more rostrally, the label adjacent to area 29 (i.e., in the supracallosal subiculum, the indusium griseum, and just dorsolateral of area 29) became increasingly light and disappeared by the caudal limit of the medial dorsal thalamic nucleus. The disappearance of this label suggests some termination within the supracallosal subiculum and indusium griseum, although it might also reflect fibers that emerged immediately dorsal to 29a–c and headed toward the region of the cingulum.

### Rhinal Plus Subicular Projections

**FIGURE 10:**
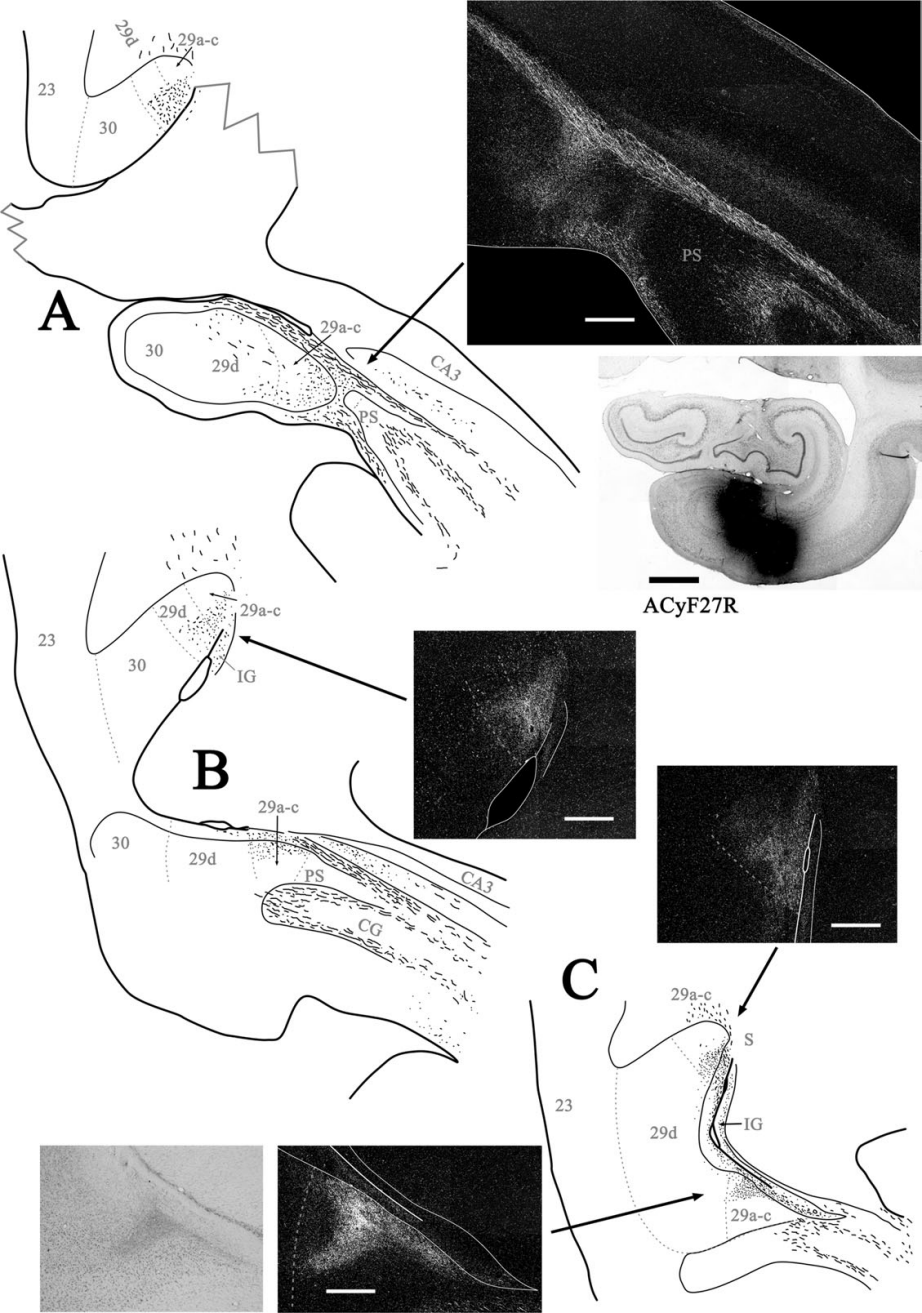
Case AF27R—rostral subiculum, presubiculum, and caudal rhinal cortex injection. Dark-field photomicrographs and line drawings of coronal sections through the posterior cingulate region showing the distribution of anterograde label, including fibers, in Case AF27R which received two injections in the rostral hippocampal formation; (1) in presubiculum and adjacent subiculum, (2) in very caudal perirhinal cortex and deep layers of adjacent entorhinal cortex. Sections A–C go from rostral to caudal. The locations of the dark-field photomicrographs are indicated by arrows. The bright field photomicrograph of Section C shows how the terminal label was concentrated in the granule cells [layer III(IV)] of area 29a–c. CG, parahippocampal cingulum; IG, indusium griseum; LV, lateral ventricle; PS, presubiculum; S, subiculum. The scale bar on the bright-field photomicrograph represents 2.0 mm, whereas that on the dark-fields represents 500 μm.

Case ACyF27R is described last as it received two injections in the rostral hippocampal formation, which together occupied the subiculum, the adjacent presubiculum, and the caudal rhinal sulcus (Figs. 4 and 10). The injection in case ACyF27R extended ∼3.2 mm in the AP plane and, as a consequence, involved the subicular cortices as well most posterior perirhinal and entorhinal cortices, with the possibility of some spread into the most rostral TH (Fig. 10). This combination of sites makes it possible to see the key features of the hippocampal and parahippocampal efferents in the same case. In case ACyF27R, one set of fibers emerged from the most caudal presubiculum to enter the caudomedial lobule (Figs. 10A,B). Some of these fibers, and others from the molecular layer of the subiculum, turned dorsally to pass just medial to the junction of the hippocampal and callosal fissures (Fig. 10). An additional, small group of fibers was found in layer I of area 30V. The final set of fibers corresponded to those seen in cases with injections in the rhinal cortex, i.e., they emerged from the caudal remnant of CA3 and passed immediately lateral to hippocampal/callosal fissure (Fig. 10C), where they were concentrated in the molecular layer of the indusium griseum. Consistent with other cases involving the rhinal cortices, *en passage* fibers were seen above area 29 of the dorsal retrosplenial cortex, where they formed a loose aggregation (Figs. 10A–C).

Terminal label within the retrosplenial cortex was restricted to area 29. In the caudomedial lobule, this label was very strongly associated with layers I and III(IV) of area 29a–c (see Figs. 10B,C). Label in the most caudal part of the dorsal retrosplenial cortex was likewise concentrated in area 29a–c [layers I and III(IV)]. The lighter label in adjacent 29d was found across layers I, III, and III(IV). Moving a little more anterior in dorsal area 29, the label again became increasingly restricted to layers I and III(IV) of 29a–c, and finally was confined to just layer I of 29a–c. This layer I label became progressively lighter in more rostral parts of the dorsal retrosplenial, until it could not be followed.

## DISCUSSION

Current understanding of the termination sites of the hippocampal projections to the primate retrosplenial cortex is essentially derived from retrograde tracer studies. The only previous anterograde tracer study (Rosene and Van Hoesen,^1977^) simply noted the existence of such projections. While retrograde tracing studies reveal much about the origins of these connections (Rosene and Van Hoesen,^1977^; Baleydier and Mauguiere,^1980^; Vogt and Pandya,^1987^; Morris et al.,1999a; Kobayashi and Amaral,^2003^; Parvizi et al.,^2006^), these studies have struggled to confine tracer injections into specific retrosplenial subregions. For this reason, this study, which placed anterograde tracer injections within the subiculum, provides new insights about termination patterns within areas 29 and 30. Data from two macaque species were combined in this study as no systematic difference could be seen between the two sets of monkeys.

Ipsilateral retrosplenial projections were found whenever the injection involved the subiculum or presubiculum, consistent with evidence from retrograde tracer studies (Rosene and Van Hoesen,^1977^; Morris et al.,1999a; Kobayashi and Amaral,^2003^; Parvizi et al.,^2006^). While the CA1 field may provide a handful of retrosplenial projections (Insausti and Munoz,^2001^), any such projections make up a very small minority of the inputs. The present findings strongly indicate that all rostro-caudal levels of the subiculum project to area 29, and that these projections consistently focus on layer I. While a part of this layer I label reflects those fibers that can be followed through the caudal subiculum and presubiculum to then reach the retrosplenial area, the gradual decrease of this label along its route in area 29 suggests termination. More clear-cut projections are found in layer III(IV) of area 29a–c, with lighter termination sometimes seen in layer III(IV) of area 29d. The relative lack of label in layer III of area 29d would seem to support the view that the cells in this layer are in reality displaced from layer III of the adjacent area 30 (see Kobayashi and Amaral,^2000^) and, hence, would contain little or no label. Only the largest hippocampal injection (ACy28) had terminal label in dorsal area 30 [layers I and III(IV)], which occasionally extended medially to reach layer III of area 23. This area 30 label was only seen when the injection involved the presubiculum, consistent with the view that the presubiculum, rather than the subiculum, principally projects to area 30 (Morris et al.,1999a). Finally, there was evidence of a light projection from the rostral subiculum to that part of the retrosplenial cortex in the lateral bank of the rostral calcarine sulcus, i.e., area 30V of Kobayashi and Amaral (2000).

Some of the above findings rely on cases where the fornix had been sectioned many months before injection of amino acids into the hippocampal region. A pertinent issue is, therefore, whether cutting the fornix interferes with the transport of amino acids from the hippocampal formation. An earlier study found that fornix transection in monkeys does not cause cell loss in the hippocampal formation (Daitz and Powell,^1954^). Consistent with this conclusion, analyses of the present monkeys showed that hippocampal cells remain capable of transporting amino acids long after fornix lesions (e.g., Aggleton et al.,^1986^). This particular capability was particularly striking in the fornix itself, where label was transported anterogradely from the hippocampus all the way to the cut section of the tract (Aggleton et al.,^1986^), and so must involve those same hippocampal neurons with axons severed by the surgery. At the same time, it is known that in rats fornix lesions produce neuroplastic responses in the hippocampus that include sprouting and in-growth fibers (e.g., Booze and Davis,^1987^; Fass and Stein,^1987^), raising the potential for aberrant connectivity patterns. Despite these concerns, the present results gave no indication that fornix surgery affected the pattern or density of projections to the retrosplenial cortex. To illustrate this point, detailed case descriptions are provided for both intact animals and for those with transected fornices.

**FIGURE 11:**
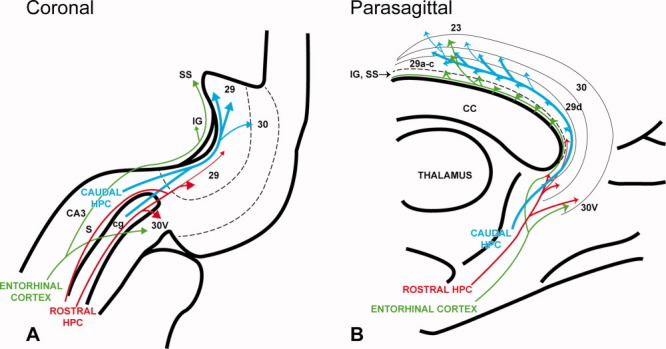
Schematic drawings illustrating the routes of the various projections from the hippocampus (subiculum) and entorhinal cortex to the retrosplenial region. A (left) depicts the coronal plane (midline to the right). The arrows show both the routes of fibers and potential sites of termination, whereas the thickness of the lines reflects the density of label. The large majority of projections from the hippocampus pass immediately medial to the merging of the hippocampal and callosal fissures. In contrast, the principal fiber route from the rhinal cortices is in the indusium griseum and, hence, is lateral to the junction of the hippocampal and callosal fissures. B (right) depicts the parasagittal plane. The stronger affinity of the hippocampal formation with area 29 than area 30 is depicted, along with the scarcity of projections to area 23. CC, corpus callosum; cg, cingulum; HPC, hippocampus; IG, indusium griseum; S, subiculum; SS, supracallosal subiculum. [Color figure can be viewed in the online issue, which is available at wileyonlinelibrary.com.]

The rostral subiculum and caudal subiculum have different termination patterns within area 29. In particular, rostral subiculum projections are largely confined to ventral area 29 in the caudomedial lobule, whereas the mid and caudal subicular regions additionally project to more dorsal portions of area 29 (Fig. 11). These finding are consistent with retrograde tracer studies (Kobayashi and Amaral,^2003^). While we found that the subicular efferents consistently target area 29 rather than area 30, the return retrosplenial projections are thought to arise from across areas 29, 30 and ventral 23 to terminate in the hippocampal formation, i.e., there is no particular affinity with area 29 (Morris et al.,1999a; Kobayashi and Amaral,^2007^). Intriguingly, these posterior cingulate efferents principally terminate in the presubiculum and parasubiculum, so that the subiculum itself receives very few reciprocal retrosplenial connections.

Hippocampal projections to dorsal area 30 arose primarily from the caudal hippocampal formation. Evidence came from finding appreciable dorsal area 30 inputs in case ACy28 (with the caveat that this case received one of the largest hippocampal injections). In contrast, no area 30 label was found following an injection into caudal prosubiculum (ACyF27L) or following a large injection involving more rostral presubiculum and subiculum (ACyF27R). This conclusion can be refined as Morris et al. (1999a) placed a retrograde tracer into dorsal area 30 and principally labeled the presubiculum and parasubiculum within the subicular region. The implication is that this area 30 projection primarily arises from the caudal presubiculum, given the lack of area 30 label in the other anterograde tracer cases. Meanwhile, the scarcity of projections to area 23 in this study agrees with prior evidence that while area 23 receives considerable projections from the parahippocampal cortex (TF,TH), scarcely any arise from the subiculum, presubiculum, or perirhinal cortex (Lavenex et al.,^2002^; Kobayashi and Amaral,^2003^).

The finding that most of the inputs to the retrosplenial cortex arise from the caudal hippocampus (Kobayashi and Amaral,^2003^) provides just one of a series of examples where the efferents from the subicular cortices show a rostral-caudal gradient (Aggleton, 2012). Hippocampal projections to sites such as the amygdala, nucleus accumbens, medial, and orbital orbital prefrontal cortices preferentially arise from the rostral subiculum (Aggleton,^1986^; Barbas and Blatt,^1995^; Carmichael and Price,^1995^; Friedman et al.,^2002^), whereas those to the mammillary bodies, anterior cingulate, and retrosplenial cortex preferentially arise from the caudal subiculum (Insausti and Munoz,^2001^; Aggleton et al.,^2005^). This commonality between the mammillary body and retrosplenial projections is notable as both sets of connections are closely associated with mnemonic processes (Tsivilis et al.,^2008^; Vann et al.,^2009^; Aggleton et al.,^2010^), both have particularly strong anatomical links with the anterior thalamic nuclei, and both arise from the subicular cortices rather than CA1.

Projections from the rhinal cortices to the retrosplenial cortex were qualitatively different to those from the subiculum (Fig. 11). These efferents often passed through the most caudal CA3 rather than the caudal subiculum or presubiculum. More caudal, these rhinal fibers were typically located lateral, not medial, to the junction of the callosal and hippocampal fissures. The resulting rhinal projections often involved the indusium griseum and supracallosal subiculum, while any label in area 29 was much lighter and typically confined to the molecular layer. The only exception was case ARhF23L, where an injection in the caudal-most entorhinal cortex resulted in additional label in rostral areas 29 and 30. This finding concurs with retrograde tracing studies showing that within the entorhinal cortex it is the most caudal portion that has the most inputs to areas 29 and 30 (Morris et al.,1999a; Kobayashi and Amaral,^2003^). The lack of projections from the perirhinal cortex to the retrosplenial cortex matches the results of another autoradiographic study (Lavenex et al.,^2002^) and, along with previous retrograde tracer studies (Morris et al.,1999a, b; Kobayashi and Amaral,^2003^), indicates that this perirhinal projection is sparse.

No evidence was found that the amygdala projects to the retrosplenial cortex. This result contrasts with the description of a light projection from the basal amygdala nuclei revealed by retrograde tracer injections involving areas 23, 30, and 31 (Buckwalter et al.,^2007^). As this study included cases with anterograde tracers centered in the basal amygdaloid nuclei (see also Amaral and Price,^1984^), injection placement is unlikely to account for the apparent discrepancy. It would appear, therefore, that this amygdala projection is very light and diffuse, and so extremely difficult to detect with autoradiography.

Comparisons with the rat brain strongly suggest that the patterns of hippocampal formation projections to the posterior cingulate/retrosplenial areas are conserved. The inputs from the rat subiculum, presubiculum, and parasubiculum principally terminate in the granular retrosplenial cortex (area 29), whereas the postsubiculum and caudal entorhinal cortex project to area 30 (Wyss and Van Groen,^1992^). As the rat postsubiculum is often regarded as part of the presubiculum (e.g., Boccara et al.,^2010^) it can be seen that there is an obvious correspondence across species. As in the monkey, there is a longitudinal gradient as the rat temporal subiculum (rostral hippocampus equivalent) innervates more caudal and more ventral area 29 (area Rga), whereas the rat septal subiculum (caudal hippocampus equivalent) innervates more rostral and more dorsal area 29 (area Rgb) (Wyss and Van Groen,^1992^; van Groen and Wyss,^1990^, ^1992^, ^2003^). Finally, in the rat there is evidence of a transverse topography whereby the rat projections to area 29 mainly arise from the distal subiculum, i.e., furthest from CA1 (Naber and Witter,^1998^). Retrograde tracer studies with monkeys suggest a similar gradient (Kobayashi and Amaral,^2003^), and the lightness of the retrosplenial label in ACyF27L (prosubiculum injection) is also consistent with this conclusion. At the same time, comparisons between ACy12 (more proximal) and ACy14 (more distal) suggest that any such gradient is gradual and that retrosplenial inputs can arise from across the transverse width of the subiculum (see also Kobayashi and Amaral,^2003^).

It is widely agreed that the borders in the monkey brain between areas 29, 30, and 23 are transitional (Vogt,^2009^), and so it might be expected that the termination patterns of these hippocampal inputs will show gradations across the various borders. Although this prediction sometimes proved correct (see ACy28), area 29 stood out within the retrosplenial cortex because of its affinity with inputs from the subiculum. Other connections in the monkey that differentiate between these retrosplenial subregions include the inputs from the medial pulvinar, dorsolateral prefrontal cortex, and parietal cortex, which are largely confined to area 30 (Baleydier and Mauguiere,^1985^; Vogt and Pandya,^1987^; Morris et al.,1999a; Kobayashi and Amaral,^2003^), and so complement the hippocampal projections to area 29. These findings point to functional differences between areas 29 and 30, despite their interconnectivity (Morris et al.,1999a; Kobayashi and Amaral,^2003^). This view is supported by recent rat studies showing that areas 29 and 30 jointly support the same overall classes of learning, e.g., spatial memory, but make different contributions (van Groen et al.,^2004^; Vann and Aggleton,^2005^; Pothuizen et al.,^2009^, ^2010^). The present findings not only point to similar, subtle differences between areas 29 and 30 in the primate retrosplenial cortex but, by virtue of its greater hippocampal inputs, reinforce the view that the retrosplenial cortex has mnemonic functions distinct from those of area 23.

## References

[b1] Aggleton JP (1986). A description of the amygdalo-hippocampal interconnections in the macaque monkey. Exp Brain Res.

[b2] Aggleton JP 2012. Multiple anatomical systems embedded within the primate medial temporal lobe: Implications for hippocampal function. Neurosci Biobehav Rev.

[b3] Aggleton JP (1985). X-ray localization of limbic structures in the cynomolgus monkey (*Macaca fascicularis*. J Neurosci Methods.

[b4] Aggleton JP, Desimone R, Mishkin M (1986). The origin, course, and termination of the hippocampo-thalamic projections in the macaque. J Comp Neurol.

[b5] Aggleton JP, O'Mara SM, Vann SD, Wright NF, Tsanov M, Erichsen JT (2010). Hippocampal—Anterior thalamic pathways for memory: Uncovering a network of direct and indirect actions. Eur J Neurosci.

[b6] Aggleton JP, Vann SD, Saunders RC (2005). Projections from the hippocampal region to the mammillary bodies in macaque monkeys. Eur J Neurosci.

[b7] Amaral DG, Price JL (1984). Amygdalo-cortical projections in the monkey (*Macaca fascicularis*. J Comp Neurol.

[b8] Amaral DG, Price JL, Pitkanen A, Carmichael ST, Aggleton JP (1992). Anatomical organisation of the primate amygdaloid complex. The Amygdala: Neurobiological Aspects of Emotion, Memory and Mental Dysfunction.

[b9] Bachevalier J, Parkinson JK, Mishkin M (1985). Visual recognition in monkeys: Effects of separate vs. combined transection of fornix and amygdalofugal pathways. Exp Brain Res.

[b10] Baleydier C, Mauguiere F (1985). Anatomical evidence of medial pulvinar connections with the posterior cingulate cortex, the retrosplenial area, and the posterior parahippocampal gyrus in monkeys. J Comp Neurol.

[b11] Baleydier C, Mauguiere F (1980). The duality of the cingulate gyrus in monkey. Neuroanatomical study and functional hypothesis. Brain.

[b12] Barbas H, Blatt GJ (1995). Topographically specific hippocampal projections target functionally distinct prefrontal areas in the rhesus monkey. Hippocampus.

[b13] Boccara CN, Sargolini F, Thoresen VH, Solstad T, Bitter MP, Moser EI, Moser M-B (2010). Grid cells in pre- and parasubiculum. Nat Neurosci.

[b14] Booze RM, Davis JN (1987). Persistence of sympathetic ingrowth fibers in aged rat hippocampus. Neurobiol Aging.

[b15] Buckwalter JA, Schumann CM, Van Hoesen GW (2007). Evidence for direct projections from the basal nucleus of the amygdala to retrosplenial cortex in the macaque monkey. Exp Brain Res.

[b16] Carmichael ST, Price JL (1995). Limbic connections of the orbital and medial prefrontal cortex in macaque monkeys. J Comp Neurol.

[b17] Daitz HM, Powell TPS (1954). Studies of the connexions of the fornix system. J Neurol Neurosurg Psychiatry.

[b18] Epstein RA, Parker WE, Feiler AM (2007). Where am I now? Distinct roles for parahippocampal and retrosplenial cortices in place recognition. J Neurosci.

[b19] Fass B, Stein DG (1987). Effects of fimbria-fornix transection and ganglioside treatments on histochemical staining for glucose-6-phosphate dehydrogenase in the lateral septum. Synapse.

[b20] Friedman DP, Aggleton JP, Saunders RC (2002). A comparison of hippocampal, amygdala, and perirhinal projections to the nucleus accumbens: A combined anterograde and retrograde tracing study in the macaque brain. J Comp Neurol.

[b21] Gainotti G, Almonti S, Betta AMD, Silveri MC (1998). Retrograde amnesia in a patient with retrosplenial tumour. Neurocase.

[b22] Greene KK, Donders J, Thoits T (2006). Topographical heading disorientation: A case study. Appl Neuropsychol.

[b23] Iaria G, Chen JK, Guariglia C, Ptito A, Petrides M (2007). Retrosplenial and hippocampal brain regions in human navigation: Complementary functional contributions to the formation and use of cognitive maps. Eur J Neurosci.

[b24] Ino T, Doi T, Hirose S, Kimura T, Ito J, Fukuyama H (2007). Directional disorientation following left retrosplenial hemorrhage: A case report with fMRI studies. Cortex.

[b25] Insausti R, Munoz M (2001). Cortical projections of the non-entorhinal hippocampal formation in the cynomolgus monkey (*Macaca fascicularis*. Eur J Neurosci.

[b26] Kobayashi Y, Amaral DG (2000). Macaque monkey retrosplenial cortex. I. Three-dimensional and cytoarchitectonic organization. J Comp Neurol.

[b27] Kobayashi Y, Amaral DG (2003). Macaque monkey retrosplenial cortex. II. Cortical afferents. J Comp Neurol.

[b28] Kobayashi Y, Amaral DG (2007). Macaque monkey retrosplenial cortex. III. Cortical efferents. J Comp Neurol.

[b29] Lavenex P, Suzuki WA, Amaral DG (2002). Perirhinal and parahippocampal cortices of the macaque monkey: Projections to the neocortex. J Comp Neurol.

[b30] Lorente de Nó R (1934). Studies on the structure of the cerebral cortex. II. Continuations of the study of the ammonic system. J Psychol Neurol (Leipzig).

[b31] Maguire EA (2001a). Neuroimaging studies of autobiographical event memory. Philos Trans R Soc London B Biol Sci.

[b32] Maguire EA (2001b). The retrosplenial contribution to human navigation: A review of lesion and neuroimaging findings. Scand J Psychol.

[b33] Morris R, Petrides M, Pandya DN (1999a). Architecture and connections of retrosplenial area 30 in the rhesus monkey (*Macaca mulatta*. Eur J Neurosci.

[b34] Morris R, Pandya DN, Petrides M (1999b). Fiber system linking the mid-dorsolateral frontal cortex with the retrosplenial/presubicular region in the rhesus monkey. J Comp Neurol.

[b35] Naber PA, Witter MP (1998). Subicular efferents are organized mostly as parallel projections: A double-labeling, retrograde-tracing study in the rat. J Comp Neurol.

[b36] Parvizi J, Van Hoesen GW, Buckwalter J, Damasio A (2006). Neural connections of the posteromedial cortex in the macaque. Proc Natl Acad Sci USA.

[b37] Paxinos G, Huang X-F, Petrides M, Toga AW (2009). The Rhesus Monkey Brain in Stereotaxic Coordinates.

[b38] Pothuizen HJ, Davies M, Aggleton JP, Vann SD (2010). Effects of selective granular retrosplenial cortex lesions on spatial working memory in rats. Behav Brain Res.

[b39] Pothuizen HJ, Davies M, Albasser MM, Aggleton JP, Vann SD (2009). Granular and dysgranular retrosplenial cortices provide qualitatively different contributions to spatial working memory: Evidence from immediate-early gene imaging in rats. Eur J Neurosci.

[b40] Rosene DL, Van Hoesen GW (1977). Hippocampal efferents reach widespread areas of cerebral cortex and amygdala in the rhesus monkey. Science.

[b41] Saunders RC, Rosene DL (1988). A comparison of the efferents of the amygdala and the hippocampal formation in the rhesus monkey. I. Convergence in the entorhinal, prorhinal, and perirhinal cortices. J Comp Neurol.

[b42] Shibata H, Yukie M (2009). Differential thalamic connections of the posteroventral and dorsal posterior cingulate gyrus in the monkey. Eur J Neurosci.

[b43] Shibata H, Yukie M, Vogt BA (2009). Thalamocingulate connections in the monkey. Cingulate Neurobiology and Disease.

[b44] Sutherland RJ, Hoesing JM, Vogt BA, Gabriel M (1993). Posterior cingulate cortex and spatial memory: A microlimnology analysis. Neurobiology of Cingulate Cortex and Limbic Thalamus: A Comprehensive Handbook.

[b45] Takahashi N, Kawamura M, Shiota J, Kasahata N, Hirayama K (1997). Pure topographic disorientation due to right retrosplenial lesion. Neurology.

[b46] Tsivilis D, Vann SD, Denby C, Roberts N, Mayes AR, Montaldi D, Aggleton JP (2008). A disproportionate role for the fornix and mammillary bodies in recall versus recognition memory. Nat Neurosci.

[b47] Valenstein E, Bowers D, Verfaellie M, Heilman KM, Day A, Watson RT (1987). Retrosplenial amnesia. Brain.

[b48] van Groen T, Kadish I, Wyss JM (2004). Retrosplenial cortex lesions of area Rgb (but not of area Rga) impair spatial learning and memory in the rat. Behav Brain Res.

[b49] van Groen T, Wyss JM (1990). Connections of the retrosplenial granular a cortex in the rat. J Comp Neurol.

[b50] van Groen T, Wyss JM (1992). Connections of the retrosplenial dysgranular cortex in the rat. J Comp Neurol.

[b51] van Groen T, Wyss JM (2003). Connections of the retrosplenial granular b cortex in the rat. J Comp Neurol.

[b52] Vann SD, Aggleton JP (2005). Selective dysgranular retrosplenial cortex lesions in rats disrupt allocentric performance of the radial-arm maze task. Behav Neurosci.

[b53] Vann SD, Aggleton JP, Maguire EA (2009). What does the retrosplenial cortex do?. Nat Rev Neurosci.

[b54] Vogt BA, Vogt BA (2009). Architecture, neurocytology and comparative organization of monkey and human cingulate cortices. Cingulate Neurobiology and Disease.

[b55] Vogt BA, Pandya DN (1987). Cingulate cortex of the rhesus monkey. II. Cortical afferents. J Comp Neurol.

[b56] Vogt BA, Pandya DN, Rosene DL (1987). Cingulate cortex of the rhesus monkey. I. Cytoarchitecture and thalamic afferents. J Comp Neurol.

[b57] Vogt BA, Vogt L, Farber NB, Bush G (2005). Architecture and neurocytology of monkey cingulate gyrus. J Comp Neurol.

[b58] Wyss JM, Van Groen TV (1992). Connections between the retrosplenial cortex and the hippocampal formation in the rat: A review. Hippocampus.

[b59] Yukie M, Shibata H, Vogt BA (2009). Temporocingulate interactions in the monkey. Cingulate Neurobiology and Disease.

